# Cholesterol mediates the effects of single and multiple environmental phenols in urine on obesity

**DOI:** 10.1186/s12944-024-02113-0

**Published:** 2024-04-29

**Authors:** Ting Yu, Yuqing Zhang, Jiali Yuan, Yue Zhang, Jing Li, Zhenyao Huang

**Affiliations:** 1 School of Public Health, Xuzhou Medical University, Xuzhou, 221004 China; 2 Key Laboratory of Human Genetics and Environmental Medicine, Xuzhou Medical University, Xuzhou, 221004 China; 3 Department of Obstetrics and Gynecology, Nanjing Maternity and Child Health Care Hospital, Women’ s Hospital of Nanjing Medical University, Nanjing, China

**Keywords:** Environmental phenols, Obesity, Cholesterol, Mixture models, Mediation models

## Abstract

**Background:**

Overweight and obesity are among the leading chronic diseases worldwide. Environmental phenols have been renowned as endocrine disruptors that contribute to weight changes; however, the effects of exposure to mixed phenols on obesity are not well established.

**Methods:**

Using data from adults in National Health and Nutrition Examination Survey, this study examined the individual and combined effects of four phenols on obesity. A combination of traditional logistic regression and two mixed models (weighted quantile sum (WQS) regression and Bayesian kernel-machine regression (BKMR)) were used together to assess the role of phenols in the development of obesity. The potential mediation of cholesterol on these effects was analyzed through a parallel mediation model.

**Results:**

The results demonstrated that solitary phenols except triclosan were inversely associated with obesity (*P*-value < 0.05). The WQS index was also negatively correlated with general obesity (β: 0.770, 95% CI: 0.644–0.919, *P*-value = 0.004) and abdominal obesity (β: 0.781, 95% CI: 0.658–0.928, *P*-value = 0.004). Consistently, the BKMR model demonstrated the significant joint negative effects of phenols on obesity. The parallel mediation analysis revealed that high-density lipoprotein mediated the effects of all four single phenols on obesity, whereas low-density lipoprotein only mediated the association between benzophenol-3 and obesity. Moreover, Cholesterol acts as a mediator of the association between mixed phenols and obesity. Exposure to single and mixed phenols significantly and negatively correlated with obesity. Cholesterol mediated the association of single and mixed environmental phenols with obesity.

**Conclusions:**

Assessing the potential public health risks of mixed phenols helps to incorporate this information into practical health advice and guidance.

**Supplementary Information:**

The online version contains supplementary material available at 10.1186/s12944-024-02113-0.

## Introduction

Obesity is a type of metabolic disease caused by dietary, genetic, and environmental disorders [1, 2]. Thus, identification of the potential risk factors for obesity is crucial for the prevention of obesity-associated health issues [3]. An increasing body of epidemiological evidence has revealed that individual environmental phenols influence the incidence and progression of obesity [4–6].

Environmental phenols, the endocrine disruptor chemicals (EDCs), including triclosan (TCS), benzophenol-3 (BP-3), and parabens (Methylparaben (MP) and Ethylparaben (EP), Propylparaben (PP) and Butylparaben (BP)), are present in consumer goods, such as preservatives, ultraviolet ray protectors, and broad-spectrum antibacterials [7–10]. The daily and almost entire life’s exposure to environmental phenols undoubtedly raised the concern about the potential risk brought by them. In fact, previous research has demonstrated detectable levels of the above environmental phenols in urine samples from a sizeable portion of Americans (U.S.), and a significant negative correlation between the concentration of urine phenols and the obesity risk in U.S. adults population [11, 12]. Triglycerides (TG), low-density lipoproteins (LDL), and high-density lipoproteins (HDL) comprise the majority of cholesterol, which is essential for the basal metabolism of living cells [13]. The intake of mono-2-ethylhexyl phthalate ultimately can lead to imbalanced cholesterol deposition and transport in the liver of mice by inducing cholesterol synthesis genes Srebp2 and Hmgcr, which are associated with adipocyte hypertrophy and cholesterol overload [14]. Modifying the cholesterol balance has a substantial effect on the adipocyte metabolism of obese animals or humans [15–17]. EDCs, cholesterol metabolism and obesity are highly correlated [18, 19]. Investigating the potential impact of environmental phenols on obesity metabolism via their influence on cholesterol levels could shed light on the role of cholesterol as a mediator between obesity and environmental phenols. Such research could enhance our comprehension of the mechanisms underlying endocrine disruptors, which are capable of effectively preventing and controlling the onset and progression of obesity.

Prior studies on the potential health effects of environmental endocrine disruptors have mostly adopted traditional single contaminant analysis methods, which may overlook the complex nonlinear and nonadditive relationships that may exist between exposure to the mixed phenol components and health outcomes [20, 21]. Combining traditional logistic regression with newly developed hybrid statistical methods robustly assesses phenotypic multicollinearity, reducing measurement error bias introduced by relying solely on a single model [22]. An unprecedented phenolic-mediated model aids in comprehending the mechanisms through which environmental phenols act on obesity [23].

Representative samples of the non-institutionalized civilian population of the U.S. from 2007 to 2012 were randomly collected and utilized for this study. Combining traditional single models and new hybrid models, we elucidated the possible effects of four environmental phenol exposures on the development of obesity from different perspectives. Furthermore, a parallel mediation model was established to explore the role of cholesterol in the association between environmental phenols and obesity.

## Materials and methods

### Population studied and data processed

**Fig. 1:**
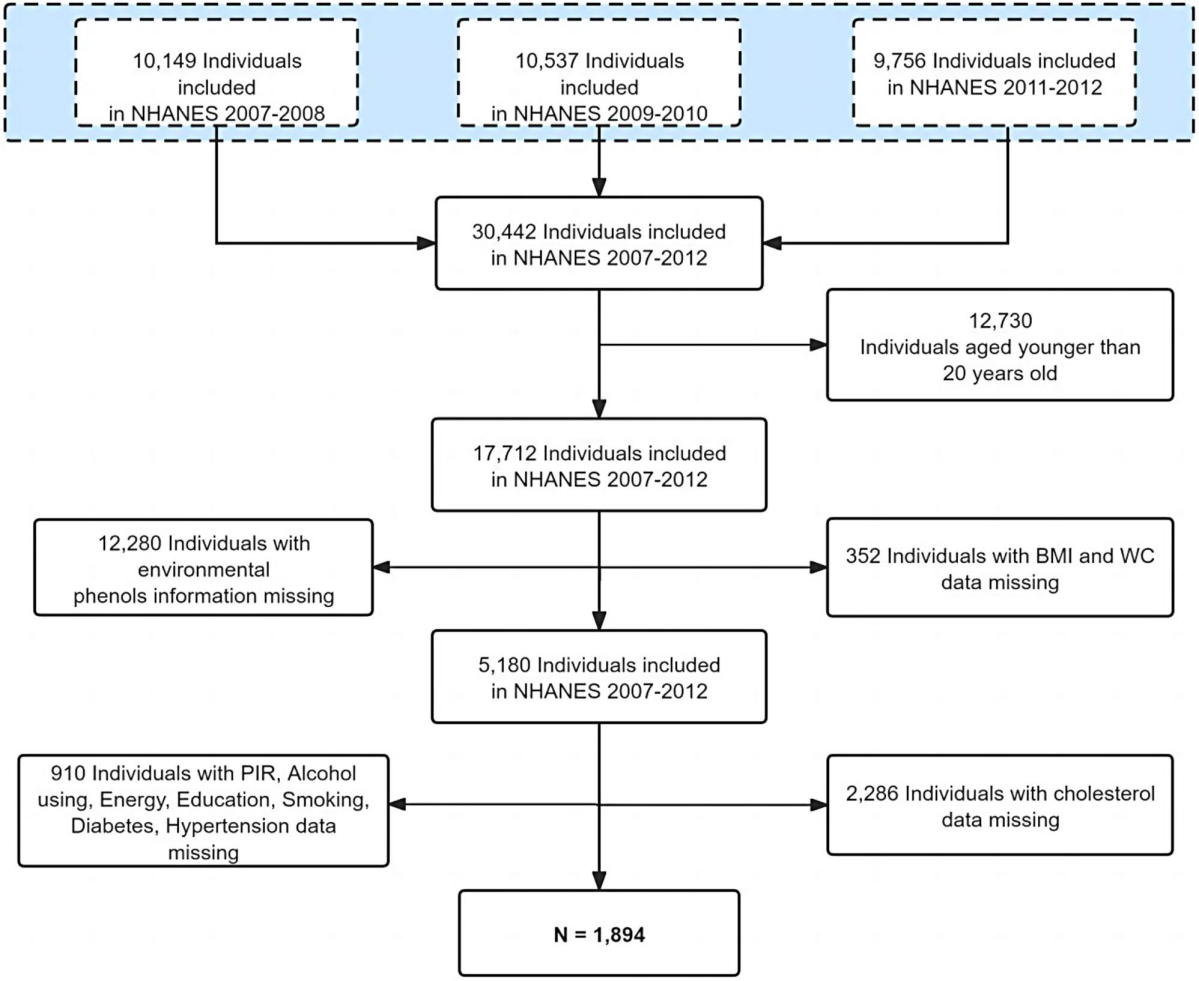
A flowchart for screening the final eligible personnel for this study. *N* = 1,894, NHANES, U.S., 2007–2012

Adults from the National Health and Nutrition Examination Survey (NHANES) who participated between 2007 and 2012 were used [24]. Besides, the analysis included 1,894 objectives with available data for TCS, BP-3, and parabens. To reduce sampling bias, some participants with missing data were excluded from the analysis: participants under the age of 20, participants with undetected environmental phenols in urine, participants without measurements of weight data (BMI and WC), participants who answered “don’t know” or were missing covariates (PIR, Alcohol using, Energy, education, Smoking, Diabetes, Hypertension), and participants with missing cholesterol data (Fig. 1).

### Measurement of BP-3, TCS, and parabens

Urinary samples from participants were collected and transported according to NHANES requirements, and environmental phenols in the urine samples were detected as soon as possible [24, 25]. NHANES provides the limit of detections (LODs) for environmental phenols, and for concentrations below the LODs, it uses the LODs divided by the square root of 2 as a substitute [26].

### Obesity assessment

General obesity are expressed as body mass index (BMI), and weight-to-height squared ratio (i.e., BMI) of more than 30 is considered to be generally obese [27, 28]. Abdominal obesity, typically assessed by waist circumference (WC), is generally defined as exceeding 102 centimeters for adult males and 88 centimeters for females [29]. https://wwwn.cdc.gov/nchs/nhanes/.

### Covariates

As age increases, so does the metabolic rate, which increases the likelihood of developing obesity; the risk of obesity differs among women and men of all ages [30]. Additionally, the prevalence of obesity differs among races as a result of cultural and environmental influences [30]. Thus, potential confounders included age (chronological age), gender, and race/ethnicity. In addition, individuals’ health behaviour is influenced by the level of education attained and the poverty-to-income ratio (PIR) [31, 32]. Smoking and excessive alcohol consumption alter the body’s metabolism and energy balance, which in turn affects body weight [33, 34]. The amount of energy intake affects the body’s fat storage [35]. For medical variables, hypertension, and diabetes may be included to minimize selection bias or survivor bias that may be introduced [36]. Urinary creatinine as a covariate was used to correct for individual urinary concentrations of phenolic metabolites. All continuous variables, except urinary creatinine, were modeled using natural transformation [24].

### Statistical analysis

#### Descriptive statistics

Continuous variables are presented as means ± standard deviations (SDs), while categorical variables are represented as percentages [37]. In the present study, the concentrations of 1,894 environmental phenols were severely right-biased; thus, log-transformed values were generated to reform the Gaussian distribution [38]. Pearson’s correlation test is a method used to measure the interactions between these substances [39].

#### Logistic regression model

Firstly, a logistic regression model was used to assess the impact of individual environmental phenol exposure. The log-transformed concentration values of these environmental phenols were sorted by four quartiles, and the model was adjusted by urinary creatinine, age, gender, race/ethnicity, PIR, education levels, physical activity, smoking status, alcohol drinking status, total energy intake, hypertension, and diabetes. Modelling of the upper three quartiles was compared with the lowest quartile (reference quartile) to derive the odds ratios (ORs) and 95% confidence intervals (CIs) [40].

#### Weighted quantile Sum (WQS)

Co-donation of multiple environmental phenols and their joint effects on obesity outcomes were considered simultaneously through WQS regression modelling [41]. This model constructs a weighted index (i.e., the WQS index) in a supervised manner, which can evaluate the overall effects of environmental exposure and the contribution of each component in mixed phenols to the overall effects [42, 43]. Here, we tested the correlation between obesity and the WQS index, as estimated according to the Quartile exposure concentration ranking (q = 4) [44]. The fitting model of WQS is as follows:

$$g\left({\upmu }\right)={\beta }_{0}+{\beta }_{1}\text{W}\text{Q}\text{S}+\text{z}{\prime } {\upphi },$$

where $g\left(\mu \right)$ indicates a nonlinear linking function that allows for generalization to continuous, binary, and other distributions; binary results were considered in this study [44, 45]. As in representative regression systems, ${\beta }_{0}$ reflects the model intercept, whereas ${\beta }_{1}$ is the regression coefficient of the weighted quantile and WQS index, that is, the overall effects of environmental phenols [46]. The WQS index is calculated as follows: WQS = $\left(\sum {w}_{i}{q}_{ij}\right)$, where ${w}_{i}$ indicates the weight of each component in the mixed environmental phenols and ${q}_{ij}$ indicates the quantile rank assigned to each subject per variable [46]. WQS was assumed that each exposed effect in the mixed phenols was in the same direction (all positive or all negative), essentially unidirectional, as it only tests for mixed effects that were positively or negatively correlated with a given result [44–46].

#### Bayesian Kernel Machine Regression (BKMR)

The BKMR model provides flexibility in modeling the combined effects of mixed phenols and elucidates the nonlinear and nonadditive associations between multiple phenols and obesity [47, 48]. The central idea is to treat the parameters in the regression model as random variables rather than as fixed but unknown values in the frequency pie statistics [47, 48]. BKMR is calculated as follows.

$$g\left({\mu }_{i}\right)=h\left({z}_{i1},\cdots ,{z}_{iM}\right)+\beta {x}_{i},$$

where$g$ denotes a monotonic link function, ${\mu }_{i}=E\left[{Y}_{i}\right]$, $h$ is a flexible function of the predictor variables ${z}_{i1},\cdots ,{z}_{iM}$, $x$ is a vector assuming a linear relationship between the covariates and the outcome, $\beta$ is the corresponding coefficient vector [47, 48], $z$ is the exposure variable, and $h(\cdot )$ is the exposure-response function [47–49]. The following procedure was used to study the cumulative toxic effects of the mixed phenols in the present study. First, we evaluated the cumulative effect by comparing the changes in obesity between all environmental phenols fixed at the 75th percentile and fixed at the 25th percentile. Subsequently, the remaining phenols were then fixed at median concentrations to obtain dose-response relationships for each metabolite with the obesity assay [50].

#### Parallel mediation analysis

To determine whether serum cholesterol mediated the associations between single and mixed environmental phenols (shown as WQS index) and obesity, we performed a parallel mediation analysis that used individual indicators as a mediator [51]. The direct effect (DE) reflects the effect of exposure to environmental phenols on obesity without a mediator, whereas the effects of exposure to environmental phenols on obesity through the mediators are considered an indirect effect (IE) [52]. The total effect (TE) represents the overall causal effect of environmental phenols on obesity [52]. Finally, the proportion of mediating effect is calculated by dividing IE by TE [52].

The covariates adjusted for WQS, BKMR, and parallel mediation model were the same as those adjusted for logistic regression. Logistic regression analysis was conducted using SPSS version 19.0 software. In R software (version 3.6.0), additional analyses including WQS regression, BKMR, and mediation regression were performed utilizing the ‘gWQS’, ‘BKMR’, and ‘mediation’ packages respectively. Statistical significance was determined at a *P*-value < 0.05 level.

## Results

### Descriptive analysis of participants

Table 1 presents the demographic characteristics of the 1894 NHANES participants collected between 2007 and 2012.

**Table 1:** Characteristics of 1,894 participants in NHANES data, 2007–2012

Characteristics	No obesity	General obesity	*P*-value	No abdominal obesity	Abdominal obesity	*P*-value
	*N* = 1,188	*N* = 706		*N* = 811	*N* = 1,083	
Age, year	48.9 ± 18.5	50.1 ± 16.5	< 0.001^a^	45.3 ± 17.8	52.3 ± 17.2	< 0.001^a^
Gender			0.002			< 0.001^a^
Male	635 (53.5)	325 (46.0)		532 (65.6)	428 (39.5)	
Female	553 (46.5)	381 (54.0)		279 (34.4)	655 (60.5)	
Ethnicity			< 0.001^a^			< 0.001^a^
Mexican American	169 (14.2)	118 (16.7)		122 (15.0)	165 (15.2)	
Other Hispanic	118 (9.9)	64 (9.1)		74 (9.1)	108 (10.0)	
Non-Hispanic White	601 (50.6)	312 (44.2)		376 (46.4)	537 (49.6)	
Non-Hispanic Black	187 (15.7)	189 (26.8)		147 (18.1)	229 (21.1)	
Others	113 (9.5)	23 (3.3)		92 (11.3)	44 (4.1)	
Education Level			0.024			0.006
Lower than high school	123 (10.4)	73 (10.3)		78 (9.6)	118 (10.9)	
High school	168 (14.1)	133 (18.8)		106 (13.1)	195 (18.0)	
Higher than high school	897 (75.5)	500 (70.8)		627 (77.3)	770 (71.1)	
PIR			0.372			0.101
≤ 1.30	366 (30.8)	234 (33.1)		237 (29.2)	363 (33.5)	
1.31–3.50	436 (36.7)	263 (37.3)		303 (37.4)	396 (36.6)	
> 3.50	386 (32.5)	209 (29.6)		271 (33.4)	324 (29.9)	
Smoking status			0.049			< 0.001^a^
Never smoker	634 (53.4)	384 (54.4)		429 (52.9)	589 (54.4)	
Past smoker	287 (24.2)	194 (27.5)		182 (22.4)	299 (27.6)	
Now smoker	267 (22.5)	128 (18.1)		200 (24.7)	195 (18.0)	
Energy			0.902			0.013
Low energy intake	508 (42.8)	309 (43.6)		335 (41.3)	481 (44.4)	
Adequate energy intake	437 (36.8)	259 (36.7)		287 (35.4)	409 (37.8)	
High energy intake	243 (20.5)	139 (19.7)		189 (23.3)	193 (17.8)	
Drink			0.067			< 0.001^a^
No	280 (23.6)	193 (27.3)		166 (20.5)	307 (28.3)	
Yes	908 (76.4)	513 (72.7)		645 (79.5)	776 (71.7)	
Diabetes			< 0.001^a^			< 0.001^a^
No	1071 (90.2)	548 (77.6)		749 (92.4)	870 (80.3)	
Borderline	23 (1.9)	19 (2.7)		12 (1.5)	30 (2.8)	
Yes	94 (7.9)	139 (19.7)		50 (6.2)	183 (16.9)	
Hypertension			< 0.001^a^			< 0.001^a^
No	845 (71.1)	347 (49.2)		620 (76.4)	572 (52.8)	
Yes	343 (28.9)	359 (50.8)		191 (23.6)	511 (47.2)	

*Note* PIR: family poverty income ratio. The data were presented as means ± standard deviations or N (%). a: *P*-value < 0.001

Briefly, 1188 (62.7%) and 706 (37.3%) objects were classified as non-generally obese and generally fat, respectively. The general obese population encompassed 325 (46.0%) men and 381 (54.0%) women with an average age of 50.1 ± 16.5, and 70.8% of the respondents had a high school education or above; the smoker and alcohol users accounted for 18.1% and 27.3%, respectively. The non-general obese population included 635 (53.5%) men and 553 (46.5%) women with an average age of 48.9 ± 18.5; Approximately 75.5% of respondents had a high school education or above. In general, obese individuals exhibited a higher average age compared to non-obese individuals, and the prevalence of obesity was higher among women than men. More hypertensive and diabetic patients were found among obese patients than among non-obese patients.

### Urinary BP-3, TCS, and paraben measurements and their correlations

The detection rates of MP, BP-3, and PP were 99.3%, 97.3%, and 93.9%, in that order; the detection rate of TCS was more 76.4%; while EP and BP were excluded from the following analyses because of their excessively low detection rates, which were 46.5% and 36.7%, respectively.

Figure S1 displays correlations among the four environmental phenols. Correlation analysis of the four interferences showed a strong correlation between MP and PP (*r* = 0.83); the next strongest correlation was between BP-3 and PP (*r* = 0.29) and TCS was weakest with BP-3, matching that of TCS and PP (*r* = 0.18). All of the correlations mentioned above reached statistical significance with a *P*-value < 0.001.

### Assessment of individual BP-3, TCS and parabens in association with obesity

The results in Table 2 showed that the concentrations of BP-3, MP, and PP were negatively correlated with obesity.

**Table 2:** Associations between BP-3, TCS, and parabens and obesity in the NHANES

	BMI		WC	
	OR (95% CI)	*P-*value	OR (95% CI)	*P-*value
BP-3		< 0.001^a^		0.013
Q1	1.00		1.00	
Q2	1.173(0.88,1.56)	0.279	1.212(0.90,1.64)	0.208
Q3	0.911(0.68,1.22)	0.537	0.937(0.69,1.27)	0.671
Q4	0.658(0.48,0.90)	0.009	0.747(0.55,1.02)	0.069
TCS		0.798		0.642
Q1	1.00		1.00	
Q2	1.011(0.76,1.35)	0.940	0.976(0.73,1.31)	0.872
Q3	1.000(0.74,1.34)	0.986	0.937(0.70,1.26)	0.669
Q4	1.041(0.78,1.40)	0.789	0.937(0.70,1.26)	0.668
MP		0.004		< 0.001^a^
Q1	1.00		1.00	
Q2	0.611(0.46,0.82)	0.001	0.747(0.56,1.01)	0.054
Q3	0.627(0.46,0.85)	0.002	0.517(0.38,0.70)	< 0.001^a^
Q4	0.618(0.45,0.85)	0.003	0.587(0.42,0.81)	< 0.001^a^
PP		< 0.001^a^		< 0.001^a^
Q1	1.00		1.00	
Q2	0.693(0.52,0.93)	0.013	0.705(0.53,0.94)	0.019
Q3	0.627(0.46,0.86)	0.003	0.521(0.38,0.71)	< 0.001^a^
Q4	0.544(0.39,0.75)	< 0.001^a^	0.545(0.39,0.76)	< 0.001^a^

*Note* Adjusted covariates: urinary creatinine, age, gender, race, family PIR, education levels, smoking status, alcohol drinking status, total energy intake, hypertension, and diabetes. CI: confidence interval. BMI: higher body mass index. WC: waist circumference. PIP: posterior inclusion probability. BP-3: benzophenol-3 (BP-3). TCS: triclosan. MP: methyl paraben. PP: propyl paraben. OR: Odd Ratio. a: *P*-value < 0.001

BP-3 was significantly and negatively associated with general obesity (*P*-value < 0.001); the Odd Ratios (ORs) of the increase over the first quartile were 1.173 (95% CI: 0.88–1.56), 0.911 (95% CI: 0.68–1.22), and 0.658 (95% CI: 0.48–0.90), respectively. MP is positively associated with general obesity (*P*-value = 0.004); the ORs of the increase over the first quartile were 0.611 (95% CI: 0.46–0.82), 0.627 (95% CI: 0.46–0.85), and 0.618 (95% CI: 0.45–0.85), respectively. PP was also negatively associated with general obesity (*P*-value < 0.001); the ORs of the increase over the first quartile were 0.693 (95% CI: 0.52–0.93), 0.627 (95% CI: 0.46–0.86) and 0.544 (95% CI: 0.39–0.75), respectively. The association between TCS and general obesity was not statistically mathematically significant (*P-*value = 0.798). A similar relationship to that of general obesity arose between the four phenols and abdominal obesity.

### Assessment of mixed BP-3, TCS and parabens in association with obesity

**Fig. 2:**
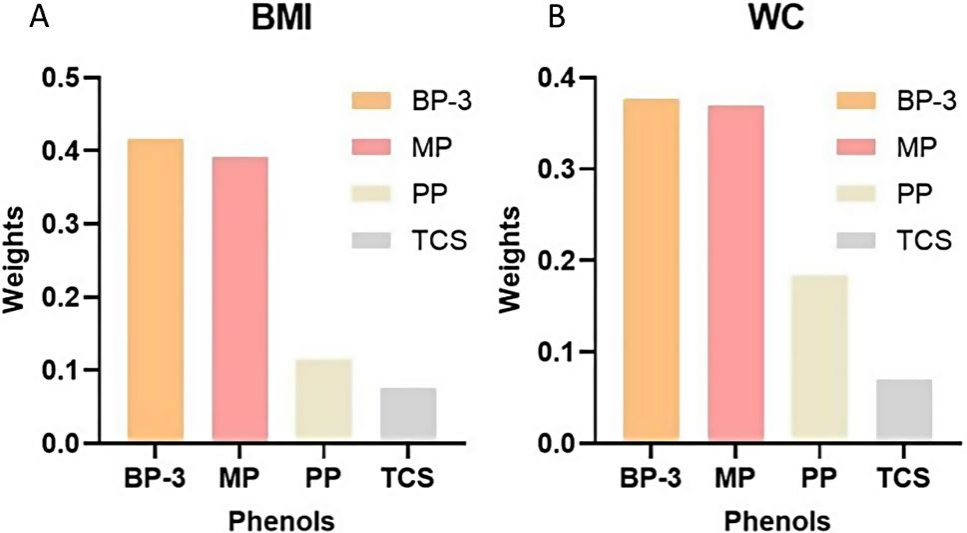
The weights of each environmental phenol in the WQS model regression index. The figure showed the weights of each phenol contributing to the overall effect. **A** WQS model regression index weights for general obesity. **B** WQS model regression index weights for abdominal obesity. The models were adjusted for urinary creatinine, age, gender, race/ethnicity, PIR, education levels, physical activity, smoking status, alcohol drinking status, total energy intake, hypertension, and diabetes

The WQS model was then used to evaluate the association between exposure to mixed phenols and obesity, and BKMR not only assessed these mixed effects but also showed the univariate exposure-response function. The WQS index, representing the mixed phenols, indicated a negative association with general obesity (OR: 0.770; 95% CI: 0.644–0.919) (Table S1). The weight order of all WQS indexes was as follows: BP-3 (41.62%), MP (39.24%), PP (11.53%), and TCS (7.61%) (Fig. 2A), indicating that BP-3 was the most predominant contributor to the effects on these measures. This negative correlation was also observed in environmental phenols and abdominal obesity (OR: 0.781; 95% CI: 0.658–0.928), and their contributions were consistent with the results of general obesity (Table S1, Fig. 2B).

**Fig. 3:**
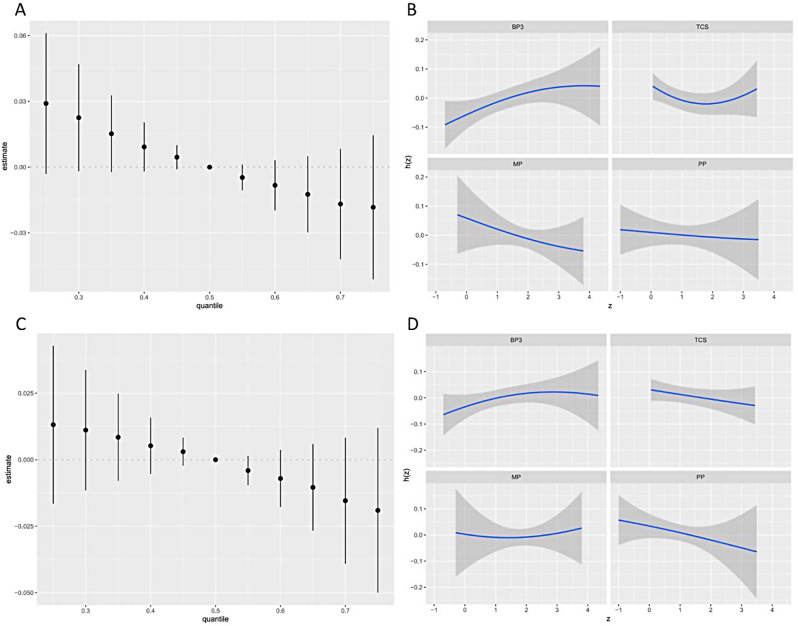
The overall effect of the mixtures on obesity and univariate exposure-response function. **A** Overall risk (95% CI) of the mixture on general obesity when comparing all the environmental phenols at different percentiles with all of them fixed at the median level by BKMR model. **B** Overall risk (95% CI) of the mixture on abdominal obesity when comparing all the environmental phenols at different percentiles with all of them fixed at the median level by BKMR model. **C** Univariate exposure-response functions for each environmental phenol on general obesity, with other metabolites fixed at their median concentrations by BKMR model. **D** Univariate exposure-response functions for each environmental phenol on abdominal obesity, with other metabolites fixed at their median concentrations by BKMR model. The models were adjusted for urinary creatinine, age, gender, race/ethnicity, PIR, education levels, physical activity, smoking status, alcohol drinking status, total energy intake, hypertension, and diabetes

The combined effects of TCS, BP-3, and parabens on general obesity were further revealed through BKMR (Fig. 3A). Compared with the medians, when the concentration of four environmental phenols was at a certain percentile, the differences between general obesity and 95% CI were used to identify the estimated overall effect. Our results indicated that the combined effects of TCS, BP-3, and parabens on general obesity were statistically significant when whole TCS, BP-3, and parabens were at or exceeded the 30th percentile. Furthermore, the higher the combined concentration of the TCS, BP-3, and paraben, the greater the effect of its negative correlation with general obesity.

Univariate exposure-response functions were estimated for TCS, BP-3, and parabens, which allows for the combination of observed data with a priori knowledge to provide more accurate and reliable results (Fig. 3B). Consistent with the results of logistic regression, the direction of exposure-response obtained through the BKMR model revealed a negative correlation of MP and PP with obesity when other phenols were fixed at their median concentrations. A reverse U-shaped association was observed between BP-3 and general obesity. A similar association exists between abdominal obesity and these same substances (Fig. 3C-D).

The posterior inclusion probability (PIP) values show the probability distribution of the effects of several phenols on obesity (Table S1).

Finally, this generalized the research results of the aforementioned three analysis models. As shown in Table S1, BP-3, MP, and PP were inversely associated with obesity, while no significant association was found between TCS and general obesity. Exposure to mixed phenols was negatively associated with obesity in the WQS regression and BKMR models. In all three models, the results for general obesity and abdominal obesity were consistent.

### 3.5 Assessment of the mediating effects of cholesterol

In addition, the parallel mediation analysis was used to identify the potential mediating role of cholesterol in the association between environmental phenols and obesity. Specifically, HDL was estimated to explain the association of BP-3, TCS, MP, and PP with general obesity, and the proportions of mediation were 34%, 44%, 29%, and 27%, respectively (all *P*-value < 0.05) (Table S1). LDL only mediated the relationship between BP-3 and general obesity with a 5% proportion of mediation (*P*-value < 0.05) (Table S1). TG was not a mediator between the association of four environmental phenols with general obesity (Table S1). The mediating effects of cholesterol on the association between individual environmental phenols and general obesity also existed in abdominal obesity (Table S4). Moreover, cholesterol parallelly mediated the associations of mixed phenols with obesity (Table 3).

**Table 3:** Cholesterol mediated the associations between the mixtures and obesity in the NHANES

Mixtures					
		TE	DE	IE	Proportion of mediation
BMI	M_1_ (TG)	-0.040(-0.057,-0.020)	-0.038(-0.055,-0.020)	-0.003(-0.005,-0.000)	7%
	M_2_ (LDL)	-0.040(-0.057,-0.020)	-0.039(-0.056,-0.020)	-0.002(-0.004,-0.000)	4%
	M_3_ (HDL)	-0.039(-0.056,-0.020)	-0.025(-0.042,-0.010)	-0.014(-0.019,-0.010)	35%
WC	M_1_ (TG)	-0.026(-0.039,-0.010)	-0.025(-0.39,-0.010)	-0.001(-0.004,-0.000)	4%
	M_2_ (LDL)	-0.027(-0.040,-0.010)	-0.025(-0.38,-0.010)	-0.002(-0.004,-0.000)	8%
	M_3_ (HDL)	-0.026(-0.040,-0.010)	-0.014(-0.28,0.000)	-0.012(-0.016,-0.010)	44%

*Note* Adjusted covariates: urinary creatinine, age, gender, race, family PIR, education levels, smoking status, alcohol drinking status, total energy intake, hypertension, and diabetes. TG: triglyceride. LDL: low density lipoprotein cholesterol. HDL: high-density lipoprotein cholesterol. TE: total effect. IE: indirect effect. DE: direct effect. BMI: higher body mass index. WC: waist circumference

**Fig. 4:**
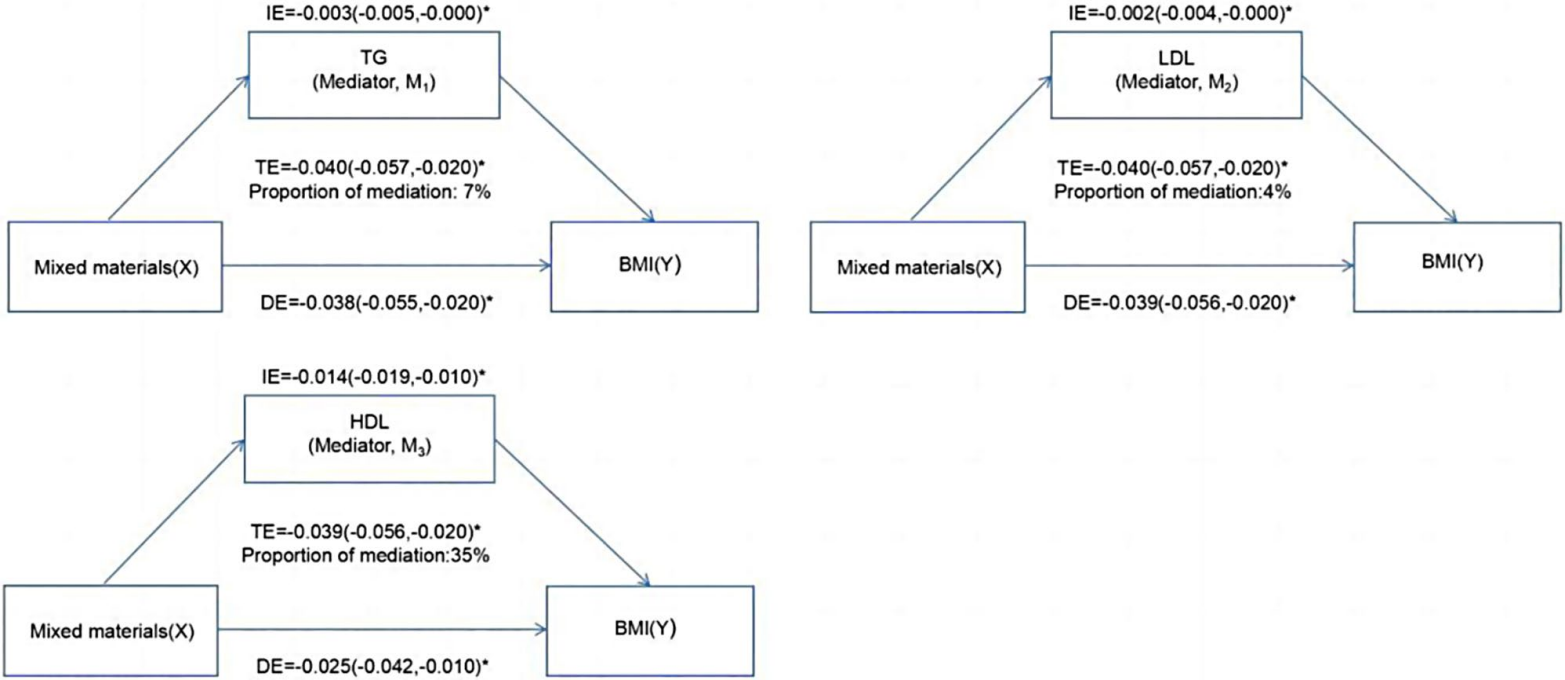
Mediation analysis of cholesterol on the interaction between mixed phenols and obesity. Cholesterol mediated association between mixed environmental phenols and general obesity. The models were adjusted for urinary creatinine, age, gender, race/ethnicity, PIR, education levels, physical activity, smoking status, alcohol drinking status, total energy intake, hypertension, and diabetes. *: *P*-value < 0.05.

TG was estimated to explain 7% of the association between mixed phenols and obesity. LDL accounted for 4% of the relationship between the mixed phenols and obesity (Fig. 4).

Figure 4 depicts that HDL elucidated 35% of the correlation between the mixed phenols and obesity. Similarly, the mediating effect of cholesterol on the mixed phenols and abdominal obesity is found in Figure S1 and Table 3.

## Discussion

The present study combines both individual and hybrid models to jointly explore the effects of exposure to individual and mixed phenols on obesity risk among a U.S. population. In logistic regression, solitary BP-3, MP, and PP were inversely associated with obesity, while no significant association was observed between single TCS and obesity. The results of both mixing models showed that mixed phenols were also negatively correlated with obesity. Notably, the aforementioned associations were mediated by cholesterol through parallel mediation analysis. HDL mediated the association between single environmental phenol and obesity, while LDL mediated only the association between BP-3 and obesity. In addition, all three types of cholesterol were mediators in a mediation model examining the role of mixed phenols. In all models, the results of abdominal obesity and general obesity were consistent, demonstrating the authenticity of our research results.

Studies have reported the inverse association of environmental phenol concentrations in urine with obesity risk. For instance, obese participants may have lower concentrations of MP and PP in their urine compared with those with a normal BMI [53]. A national biological monitoring survey conducted in Canada showed a negative correlation between methylparaben and female obesity [54]. In addition, a cross-sectional study based on Korean adults found that the ORs for obesity showed a decrease in the highest quartiles of certain endocrine-disrupting substances detected in the urine [55]. Attractively, both the mixed model Sparse Decompositional Regression and BKMR found a mixed negative contribution of BP-3, MP, and PP to obesity [22, 56]. Overall, the aforementioned evidence supports our view that there was a negative correlation between BP-3, TCS, and parabens and obesity. Phthalate deposition in human fat pools may be attributed to the lipophilic nature of endocrine-disrupting environmental substances, which may be helpful in explaining our research findings [57, 58]. In addition, endocrine disruptors may affect the expression of endogenous cannabinoids and cannabinoid receptors, thereby altering the expression of leptin or neuropeptide Y, the signaling neuropeptide for fatty liver [59]. Finally, endocrine disruptors may also affect the expression of lipid metabolism-related enzymes, transcription factors, and adipocytokines [59]. On the one hand, urinary TCS has been found to have a significant negative correlation with BMI and waist circumference in U.S. children and adults [60]. On the other hand, TCS was also reported to be positively associated with adiposity measurements conducted on girls who were overweight at baseline [61]. Our results did not reveal a connection between TCS and obesity as only adults were included as participants, and the genetic effect of obesity was not considered in the present study. Further and more comprehensive analysis was necessary. Consistent with our research findings, Xue and fellows did not find an association between TCS exposure and obesity [62].

The relationship between endocrine disruptors and lipids is very complex. Low concentrations of the same chemicals can increase fat production, whereas high concentrations can inhibit fat cell differentiation [63]. Reduced safe storage locations of obesity lipophilic chemicals could potentially render them more hazardous in nature compared with agents that induce obesity [64]. Although the association between these persistent organic compounds and obesity cannot be fully predicted, hybrid models can at least better identify interactions between homologous chemicals [49]. More common are studies looking at how individual environmental exposures affect health. However, chemical exposure invariably results in the manifestation of mixture effects. Patterns of exposure to mixed environmental phenols and the potential effects of mixed exposure on obesity are unknown. BP-3, TCS, MP, and PP are often combined in products, and there was a strong correlation between them (*P*-value < 0.05) [63]. Therefore, analyzing the relationship between mixed environmental phenols and obesity might provide us with a more practical perspective for understanding the synergistic effects of these chemicals. The WQS model reflected the combined effects of mixed exposure and explained the contributions of each component in the mixed effects [45, 46]. Our results of the WQS model indicated a negative correlation between environmental multi-phenols and obesity, with BP-3 contributing the most to this association. One limitation of WQS is the reduced statistical power caused by the need to split the dataset into training and validation sets, which may also lead to unrepresentative datasets and unstable parameter estimation [46]. Thus, BKMR was further used, as it does not need to set parameter expression forms, allowing for the existence of nonlinear effects and interactions [43, 44]. The BKMR model can also generate kernel functions based on the mixture variables included in the model. Bayesian sampling and analysis methods can be used to generate the association curve between the mixed phenol components and the disease variables included in the model [43, 44]. With this method, we found the negative association of mixed environmental phenols with obesity, consistent with the results of the WQS model.

Understanding the effects and mechanisms of action of EDCs on lipid metabolism is important for a comprehensive assessment of the health risks of EDCs. Mechanistically, it has been shown that EDCs can directly increase the number of adipocytes by upregulating the expression of genes that promote adipocyte production [65]. EDCs can also indirectly increase fat content by disrupting metabolic pathways, altering metabolic set points, inducing adverse changes in the gut microbiome, and upregulating obesogenic diets [66]. Cholesterol imbalance is a feature of enlarged fat cells in obese states, and cholesterol normalization is beneficial in reversing insulin resistance and combating the development of obesity [15]. Previous investigations have also documented the effect of EDCs on cholesterol homeostasis [67–69]. Parallel mediation analyses were used to explore whether cholesterol plays a mediating role, our results indicated that the association between single/mixed phenols and obesity was mediated or at least mediated in part by different cholesterol types. This study assesses their combined effects on health outcomes through single and mixed chemical models, providing new ideas for real-life exposure prevention and treatment strategies and new evidence for future epidemiological and toxicological studies [56].

## Study strengths and limitations

The present study used WQS and BKMR model to solve nonlinear and data imbalance problems that cannot be handled through logistic regression [70]. The BKMR method handles parameter uncertainty well and provides more comprehensive and accurate inference results, especially excelling in the face of small sample data or incomplete data [47, 48]. The WQS and BKMR hybrid models were used to determine weights based on self-help sampling experience, which better reflects the complexity of real-life endocrine disruptor exposure [56, 70]. These two hybrid models can be applied in various environmental health studies for better exposure effect analysis, risk assessment, and exploration of factor interactions [56, 70].

However, this study has some shortcomings. First, the results of parallel mediation may not fully explain the mediating effect of cholesterol, as studies on the interaction between the four types of cholesterol have not been considered [52]. Additionally, the WQS model cannot assess the combined effects of phenolics in different directions of effect but can only evaluate the effects of phenolics acting in a single direction separately [71, 72]. Another limitation is that The NHANES data provide static urine sample data and lack information on dynamic changes in biomarkers [52, 56]. We have difficulty assessing changes and trends in health factors over time, limiting research on causal associations between environmental phenols and obesity [52, 56].

## Conclusions

In summary, the results of this study show a negative correlation between single and mixed environmental phenols and an increased risk of obesity, with identical results for abdominal obesity and general obesity. Furthermore, mediation analysis revealed that the association between single and mixed environmental phenols and obesity risk may be mediated by cholesterol. These results suggest that the combined effects of mixed chemicals provide a better description of their true toxicity than single chemical exposure assessments, emphasizing the need for incorporating mixed phenols into chemical testing and risk assessment processes.

### Electronic supplementary material

Below is the link to the electronic supplementary material.

Supplementary Material 1

## Data Availability

No datasets were generated or analysed during the current study.

## Abbreviations

BP-3: Benzo Phenone-3
BP: Butylparaben
BMI: Body Mass Index
BKMR: Bayesian Kernel Machine Regression
CI: Credible Interval
DE: Direct Effect
EP: Ethylparaben
EDC: Endocrine Disruptor Chemical
HDL: High-Density Lipoprotein
IE: Indirect Effect
KMR: Kernel Machine Regression
LDL: Low-Density Lipoprotein
LOD: Limit Of Detection
MP: Methylparaben
NHANES: National Health and Nutrition Examination Survey
OR: Odds Ratio
PP: Propylparaben
PIR: Poverty Income Ratio
PIP: Posterior Inclusion Probability
SD: Standard Deviation
TCS: Triclosan
TG: Triglycerides
TE: Total Effect
U.S.: Americans
WC: Waist-Circumference
WQS: Weighted Quantile Sum
