# Supplementary material for: Cholesterol mediates the effects of single and multiple environmental phenols in urine on obesity
Source: Lipids Health Dis. 2024 Apr 29;23:126. doi: 10.1186/s12944-024-02113-0 (PMC11057097; doi:10.1186/s12944-024-02113-0)
Supplement: Supplementary file 1 — Supplementary Material 1 [file 12944_2024_2113_MOESM1_ESM.docx]

**Supplementary material**

**Figure legends**

**Figure S1. Correlations of the environmental phenols (BP-3, TCS, MP, PP).** Evaluating the correlation between four environmental phenols using Pearson test. ***: *P-*value < 0.001.

**Figure S2. Mediation analysis of cholesterol on the interaction between phenol mixtures and obesity.** Cholesterol mediated association between mixed environmental phenols and abdominal obesity. The models were adjusted for urinary creatinine, age, gender, and race/ethnicity, family income-to-poverty ratio (PIR), education levels, physical activity, smoking status, alcohol drinking status, total energy intake, hypertension, and diabetes. *: *P-*value < 0.05.

## **Figure S1**


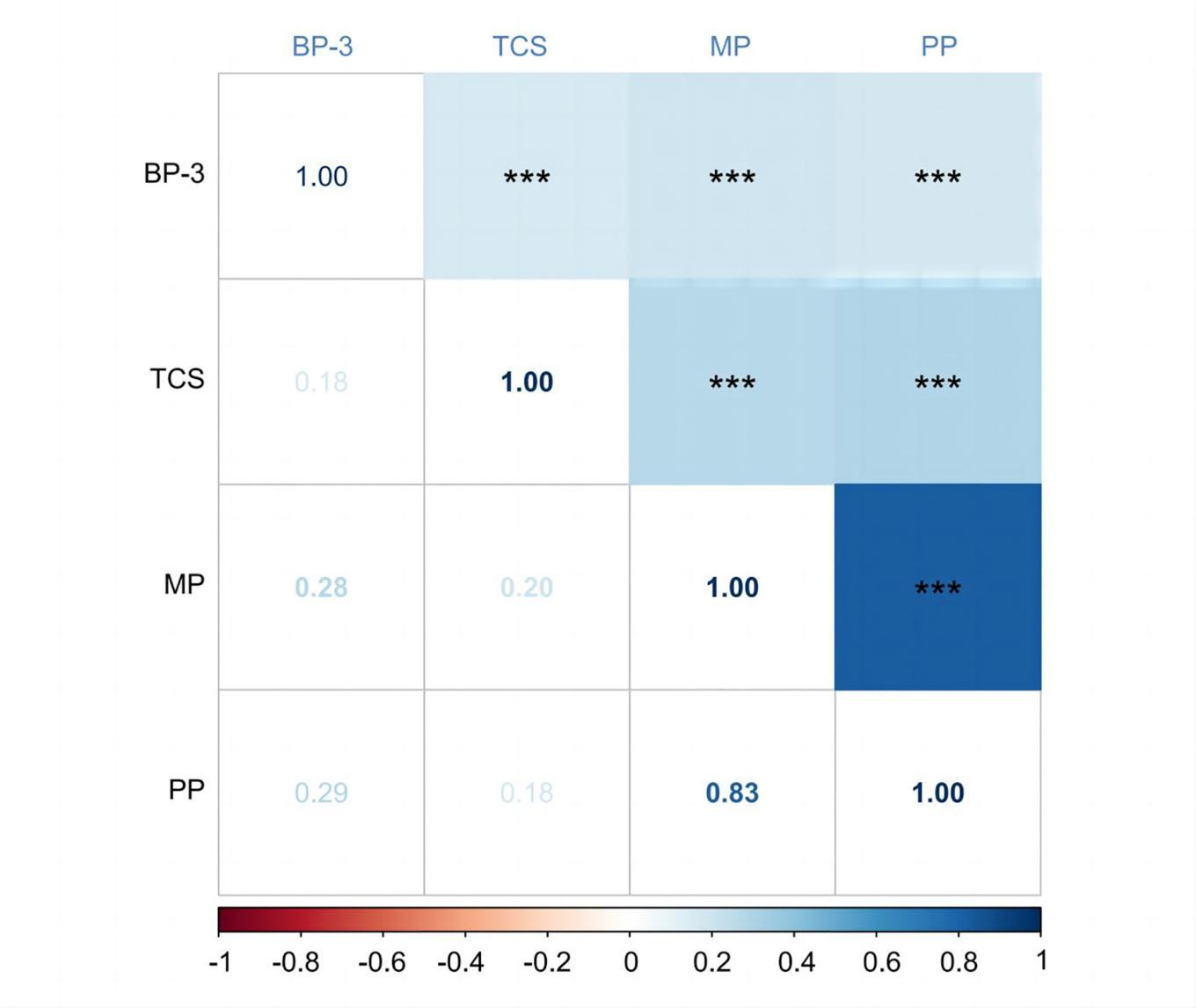


## **Figure S2**


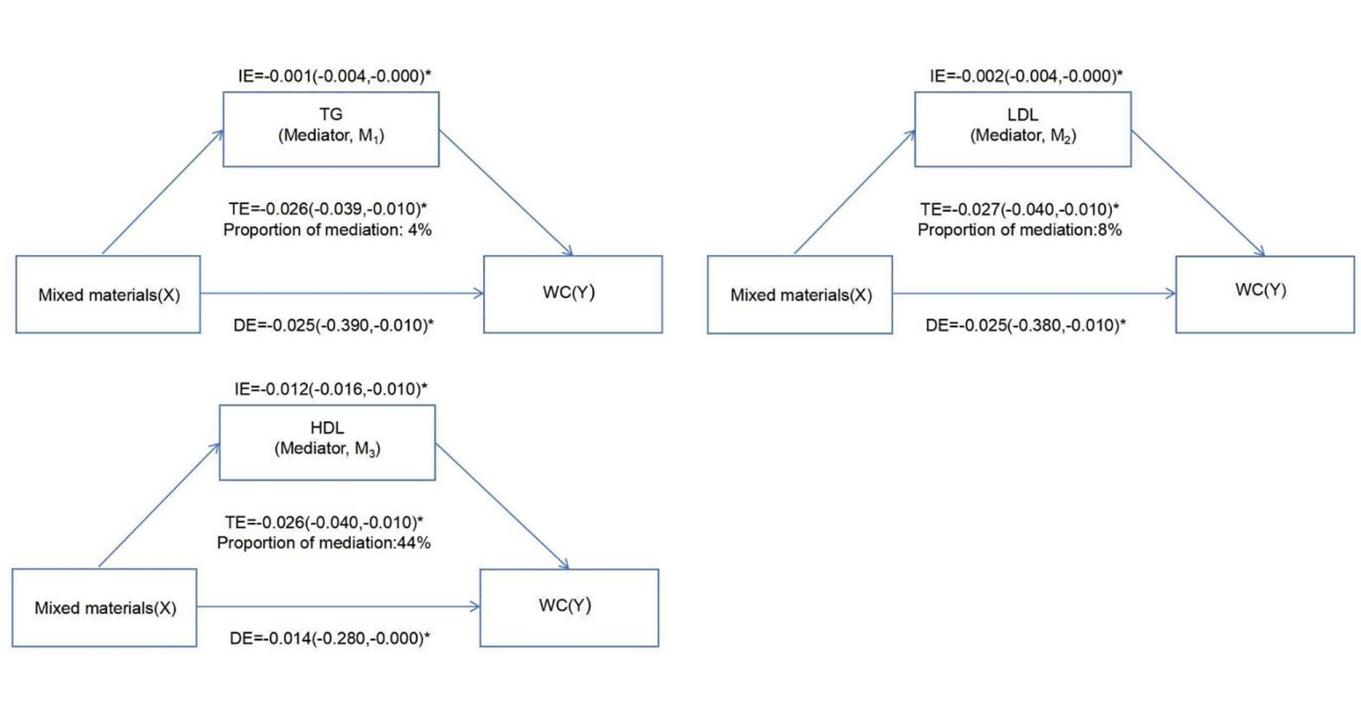


## **Table S1 WQS model was used to evaluate the relationship between WCS index and obesity.**

|  | BMI |  | WC |  |
| --- | --- | --- | --- | --- |
| Outcomes | OR (95% CI) | *P-*value | OR (95% CI) | *P-*value |
| b1_negative |  |  |  |  |
|  | 0.770(0.644,0.919) | 0.004 | 0.781 (0.658,0.928) | 0.004 |

**Note:** Adjusted covariates: urine creatinine, age, gender, race, family PIR, education levels, smoking status, alcohol drinking status, total energy intake, hypertension, and diabetes. CI: confidence interval. OR: Odd ratio. BMI: higher body mass index. WC: waist circumference.

## **Table S2 In BKMR, the PIP value of various BP-3, TCS, parabens.**

|  | BMI | WC |  |
| --- | --- | --- | --- |
| Variable | PIP | PIP |  |
| BP-3 | 0.593 | 0.090 |  |
| TCS | 0.466 | 0.125 |  |
| MP | 0.718 | 0.121 |  |
| PP | 0.283 | 0.197 |  |

**Note**: Adjusted covariates: urinary creatinine, age, gender, race, family PIR, education levels, smoking status, alcohol drinking status, total energy intake, hypertension, and diabetes. BMI: higher body mass index. WC: waist circumference. PIP: posterior inclusion probability. BP-3: benzophenol-3 (BP-3). TCS: triclosan. MP: methyl paraben. PP: propyl paraben.

## **Table S3 Cholesterol mediated the relationship between BP-3, TCS, Parabens and obesity in NHANES.**

|  |  | TE | | DE | | IE | proportion of mediation |
| --- | --- | --- | --- | --- | --- | --- | --- |
|  | BP-3 | -0.0207(-0.0316,-0.0084) | -0.0194(-0.0296,-0.0073) | | -0.0014(-0.0034,0.0006) | |  |
| M_1_(TG) | TCS | 0.0003(-0.0105,0.0109) | -0.0004(-0.0115,0.0099) | | 0.0007(-0.0013,0.0026) | |  |
|  | MP | -0.0267(-0.0404,-0.0143) | -0.0254(-0.0391,-0.0125) | | -0.0013(-0.0037,0.0011) | |  |
|  | PP | -0.0224(-0.0329,-0.0120) | -0.0207(-0.0310,-0.0102) | | -0.0017(-0.0037,0.0001) | |  |
| M_2_(LDL) | BP-3 | -0.0208(-0.0313,-0.0098) | -0.0197(-0.0304,-0.0089) | | -0.0011(-0.0024,-0.0002) | | 5% |
|  | TCS | -0.0001(-0.0102,0.0103) | -0.0003(-0.0102,0.0102) | | 0.0002(-0.0006,0.0012) | |  |
|  | MP | -0.0266(-0.0395,-0.0125) | -0.0258(-0.0387,-0.0117) | | -0.0008(-0.0022,0.0002) | |  |
|  | PP | -0.0227(-0.0329,-0.0123) | -0.0219(-0.0320,-0.0115) | | -0.0007(-0.0019,0.0000) | |  |
| M_3_(HDL) | BP-3 | -0.0207(-0.0318,-0.0102) | -0.0138(-0.0245,-0.0035) | | -0.0069(-0.0104,-0.0034) | | 34% |
|  | TCS | -0.0003(-0.0117,0.0099) | 0.0048(-0.0057,0.0151) | | -0.0052(-0.0087,-0.0020) | | 44% |
|  | MP | -0.0259(-0.0393,-0.0126) | -0.0185(-0.0308,-0.0060) | | -0.0074(-0.0116,-0.0033) | | 29% |
|  | PP | -0.0217(-0.0315,-0.0118) | -0.0159(-0.0257,-0.0065) | | -0.0059(-0.0091,-0.0028) | | 27% |

**Note:** Adjusted covariates: urinary creatinine, age, gender, race, family PIR, education levels, smoking status, alcohol drinking status, total energy intake, hypertension, and diabetes. TG: triglyceride. LDL: low density lipoprotein cholesterol. HDL: high-density lipoprotein cholesterol. TE: total effect. IE: indirect effect. DE: direct effect. BMI: higher body mass index. WC: waist circumference.

## **Table S4 Cholesterol mediated the relationship between BP-3, TCS, Parabens and WC in NHANES.**

|  |  | TE | DE | IE | proportion of mediation |
| --- | --- | --- | --- | --- | --- |
|  | BP-3 | -0.0126(-0.0220,-0.0017) | -0.0112(-0.0202,-0.0004) | -0.0015(-0.0036,0.0006) |  |
| M_1_(TG) | TCS | -0.0037(-0.0138,0.0069) | -0.0045(-0.0148,0.0058) | 0.0008(-0.0015,0.0030) |  |
|  | MP | -0.0260(-0.0353,-0.0168) | -0.0248(-0.0343,-0.0149) | -0.0013(-0.0036,0.0011) |  |
|  | PP | -0.0219(-0.0309,-0.0124) | -0.0200(-0.0288,-0.0107) | -0.0019(-0.0039,0.0001) |  |
| M_2_(LDL) | BP-3 | -0.0131(-0.0223,-0.0031) | -0.0114(-0.0206,-0.0017) | -0.0017(-0.0033,-0.0004) | 13% |
|  | TCS | -0.0044(-0.0138,0.0060) | -0.0047(-0.0139,0.0058) | 0.0003(-0.0010,0.0019) |  |
|  | MP | -0.0263(-0.0351,-0.0154) | -0.0251(-0.0337,-0.0142) | -0.0012(-0.0028,0.0003) |  |
|  | PP | -0.0220(-0.0307,-0.0126) | -0.0209(-0.0295,-0.0114) | -0.0012(-0.0027,0.0001) |  |
| M_3_(HDL) | BP-3 | -0.0124(-0.0224,-0.0029) | -0.0064(-0.0160,0.0031) | -0.0060(-0.0089,-0.0030) | 48% |
|  | TCS | -0.0040(-0.0148,0.0064) | 0.0006(-0.0095,0.0113) | -0.0047(-0.0078,-0.0018) | 66% |
|  | MP | -0.0255(-0.0346,-0.0151) | -0.0197(-0.0289,-0.0093) | -0.0058(-0.0090,-0.0026) | 23% |
|  | PP | -0.0214(-0.0302,-0.0124) | -0.0163(-0.0249,-0.0076) | -0.0051(-0.0077,-0.0025) | 24% |

**Note:** Adjusted covariates: urinary creatinine, age, gender, race, family PIR, education levels, smoking status, alcohol drinking status, total energy intake, hypertension, and diabetes. TG: triglyceride. LDL: low density lipoprotein cholesterol. HDL: high-density lipoprotein cholesterol. TE: total effect. IE: indirect effect. DE: direct effect. BMI: higher body mass index. WC: waist circumference.
